# Age-Related Macular Degeneration: A Connection between Human Herpes Virus-6A-Induced CD46 Downregulation and Complement Activation?

**DOI:** 10.3389/fimmu.2017.01314

**Published:** 2017-10-17

**Authors:** Walter Fierz

**Affiliations:** ^1^labormedizinisches zentrum Dr Risch, Vaduz, Liechtenstein

**Keywords:** human herpes virus-6A, age-related macular degeneration, CD46, complement system proteins, autophagy, parainflammation, inflammaging

## Abstract

Viruses are able to interfere with the immune system by docking to receptors on host cells that are important for proper functioning of the immune system. A well-known example is the human immunodeficiency virus that uses CD4 cell surface molecules to enter host lymphocytes and thereby deleteriously destroying the helper cell population of the immune system. A more complicated mechanism is seen in multiple sclerosis (MS) where human herpes virus-6A (HHV-6A) infects astrocytes by docking to the CD46 surface receptor. Such HHV-6A infection in the brain of MS patients has recently been postulated to enable Epstein–Barr virus (EBV) to transform latently infected B-lymphocytes in brain lesions leading to the well-known phenomenon of oligoclonal immunoglobulin production that is widely used in the diagnosis of MS. The cellular immune response to HHV-6A and EBV is one part of the pathogenic mechanisms in MS. A more subtle pathogenic mechanism can be seen in the downregulation of CD46 on astrocytes by the infecting HHV-6A. Since CD46 is central in regulating the complement system, a lack of CD46 can lead to hyperactivation of the complement system. In fact, activation of the complement system in brain lesions is a well-known pathogenic mechanism in MS. In this review, it is postulated that a similar mechanism is central in the development of age-related macular degeneration (AMD). One of the earliest changes in the retina of AMD patients is the loss of CD46 expression in the retinal pigment epithelial (RPE) cells in the course of geographic atrophy. Furthermore, CD46 deficient mice spontaneously develop dry-type AMD-like changes in their retina. It is also well known that certain genetic polymorphisms in the complement-inhibiting pathways correlate with higher risks of AMD development. The tenet is that HHV-6A infection of the retina leads to downregulation of CD46 and consequently to hyperactivation of the complement system in the eyes of susceptible individuals.

## Introduction

Many microorganisms use a survival strategy based on their interference with the immune system of their hosts. One way to do so is to acquire immune regulatory proteins from the host that subsequently protect them from immune-mediated attacks by the host. Examples are human immunodeficiency virus (HIV) (1) and cytomegalovirus (2) that both incorporate host cell-derived complement control proteins like CD55 and CD59 to protect themselves against complement attacks by the host. Similarly, certain *Borrelia burgdorferi* strains arm themselves with the complement regulatory proteins FHL-1/reconectin and Factor H by using complement regulators acquiring surface proteins (3). Another strategy is to use cell surface receptors of host immune cells for infection and thereby directly interfering with immune functions. A well-known example is HIV that infects host T-helper cells using the CD4 receptor (4, 5).

Other pathogenic effects can be seen when viruses infect host cells and thereby change the cell functions without killing the cells in the process. In multiple sclerosis (MS), e.g., human herpes virus-6A (HHV-6A) infects astrocytes in the brain by docking to the CD46 molecules (6–11). One effect of such HHV-6A infection in MS patients has recently been postulated to interfere with Epstein–Barr virus (EBV) in latently infected B-cells in brain lesions (12). Consequently, B-cells would be transformed by EBV and produce clonal immunoglobulins that are common in MS patients and are used as diagnostic markers in the cerebrospinal fluid. In addition, cellular immune responses to HHV-6A and EBV would induce and sustain the inflammatory lesions in MS brains. Furthermore, the infection of astrocytes with HHV-6A also leads to downregulation of the receptor CD46 that was used for entering the cell (8). Since CD46 is important in limiting the activity of the complement system, the downregulation of CD46 leads to hyperactivity of complement (13). In recent years, it has become clear that complement activity in the brain itself is an important factor in the pathogenesis of MS (14).

Based on these observations, it is postulated here that similar HHV-6A/CD46/complement interactions are central in the development of age-related macular degeneration (AMD). In this article, pathogenic mechanisms in AMD as they are known today are summarized and then a link to HHV-6A *via* CD46 is proposed. Finally, the relation of AMD to MS and other diseases where HHV-6A infection plays a pathogenic role is explored.

## Hypothesis

Age-related macular degeneration, a degenerative disease of the retina, is the leading cause of irreversible central blindness in elderly people [for review, see Ref. (15)]. Although many risk factors are known [for review, see Ref. (16)], the etiology of AMD remains elusive. Based on known pathogenic mechanisms described below, it is proposed that HHV-6A is an etiologic agent for AMD.

### Inflammation/Parainflammation/Inflammaging

Inflammation plays an important role in the pathogenesis of AMD [for review, see Ref. (17–20)]; however, the exact inflammatory mechanisms involved remain unclear. Individuals with elevated C-reactive protein, a general systemic marker for inflammation, carry a higher risk of developing AMD (21). Locally in the retina, proinflammatory macrophages (M1) are enriched at the expense of scavenging and anti-inflammatory M2 macrophages (22). A chronic low-grade inflammation, called parainflammation, is generally considered a beneficial response to chronic insults also in AMD (23). A chronic, parainflammation characteristic for aging is called inflammaging [for review, see Ref. (24)]. Similar to age-related diseases in other organs, inflammaging is supposed to manifest itself also in AMD (25, 26).

### Complement and CD46

A central role in the inflammatory pathogenesis of AMD is accredited to the regulation of the complement system [for review, see Ref. (27, 28)]. The strong genetic risk conferred by a polymorphism of complement factor H (29–33), but also polymorphisms of ARMS2/HTRA1 (34) support this notion. At present, the function of the ARMS2 protein and the biological consequences of the polymorphism are not completely unraveled, but it has recently been found that ARMS2 functions as surface complement regulator and that ARMS2 is involved in complement-mediated clearance of cellular debris (35).

The spectrum of complement activation in the retina of AMD patients ranges from beneficial to detrimental. Therefore, complement regulation plays a key role in the pathogenesis of AMD. Membrane cofactor protein (MCP, CD46) is a well-known regulatory membrane protein that guards cells from complement attack [for review, see Ref. (36)]. CD46 acts as a cofactor for complement factor I, which protects autologous cells against complement-mediated injury by cleaving C3b and C4b deposited on the cells surface. An intergenic single-nucleotide polymorphism just 3′ of complement factor I on chromosome 4 is indeed associated with risk of advanced AMD (37).

The importance of the complement regulatory CD46 is demonstrated by the finding that retinal pigment epithelial (RPE) cells lose their CD46 expression very early in the development of geographic atrophy even before any morphological change of RPE (38). The loss of CD46 makes the RPE vulnerable to complement. Furthermore, an additional role of CD46 in RPE seems to lay in the adhesion of the RPE to its basement membrane and Bruch’s membrane, thereby safeguarding its integrity (39). The key pathogenic role of CD46 loss in AMD is also demonstrated by an experimental animal model in which *Cd46^−/−^* knockout mice develop a dry-type AMD-like phenotype (40).

### Autophagy and CD46

Autophagy is a hot topic in AMD research (41) and it is likely to play an important role in the pathogenesis of AMD as a highly regulated clearance and recycling mechanism of cytoplasmic contents [for review, see Ref. (42, 43)]. Transforming growth factor β activated kinase 1 (TAK1), a key player in the regulation of autophagy, maintains the normal function of RPE cells (44, 45). Recently, it was discovered that autophagy is triggered, when pathogens are bound to CD46 (46). This may be a cellular adaptation to infection *via* CD46.

### Chemokines and Chemokine Receptors

Associations of polymorphisms in the gene of chemokine (C-X3-C motif) receptor 1 (*CX3CR1*) with AMD susceptibility have been reported in several studies (47–49). The CX3CR1 polymorphisms result in decreased affinity for its ligand (CX3CL1, fractalkine), which in turn negatively affects microglial and macrophage migration (50). A chemotactic cytokine, RANTES or CCL5, produced by RPE cells also seems to regulate inflammatory cell migration (51).

### HHV-6A: Regulating Complement, Autophagy, and Chemokines

Human herpes virus-6A uses the membrane protein CD46 as a receptor to enter cells (8, 52, 53). Such infection is followed by downregulation of CD46. Other viruses, like measles virus (CD46), HIV (CD4), and EBV (CD21), also follow similar strategies of receptor downregulation after infection (54). The CD46 downregulation by HHV-6A may functionally impair the protective effect of CD46 against the activation of autologous complement and the consequent cellular damage as shown *in vitro* using measles virus (55). In this way, HHV-6A would interfere with key pathogenic complement mechanisms in AMD when RPE cells are infected *via* CD46.

HHV-6 infection can also impair Toll-like receptor signaling by reducing TAK1 activity as shown in infected dendritic cells (56). When RPE cells are infected with HHV-6A, the essential role of TAK1 for maintaining normal function of RPE cells through regulation of autophagy would be impaired (44, 45).

HHV-6 expresses its own chemokine receptors encoded by the U12, and U51 genes. The open reading frame U12 functionally encodes a calcium-mobilizing receptor for the β-chemokines RANTES, MIP-1α and -1β, and MCP-1 (57, 58), thereby potentially interfering with RANTES regulation of inflammatory cell migration (51). In epithelial cells already secreting RANTES, U51 expression results in specific transcriptional downregulation of the cytokine (59).

Altogether, HHV-6A, infecting RPE cells *via* CD46, would have the potential to interfere on several levels with the parainflammatory mechanisms central to AMD pathogenesis.

### Association of AMD with HHV-6A-Related Diseases

If HHV-6A has an etiologic role in the development of AMD, as hypothesized here, a higher prevalence of AMD would be expected in other diseases where HHV-6A infection is observed. In contrast to HHV-6B, which is the infectious agent of roseola in childhood, no definite clinical picture of acute HHV-6A infection could be established so far (60–62). On the other hand, HHV-6A infection has been associated with several chronic diseases like AIDS, Hashimoto’s thyroiditis, and MS and their epidemiological association is summarized in Table 1.

**Table 1:** Association of human herpes virus-6A (HHV-6A) and age-related macular degeneration (AMD) with AIDS, Hashimoto’s thyroiditis (HT), and multiple sclerosis (MS).

Disease	Association with HHV-6A	Association with AMD
AIDS	HIV-1 infection is epidemiologically associated with HHV-6A infection (63, 64). Particularly, HHV-6 is found in demyelinating lesions in AIDS patients with cerebral involvement (65) and HIV-1 and HHV-6 antigens and transcripts are found in the retina of patients with AIDS (66)	Persons with AIDS appear to have an approximately 4-fold increased prevalence of intermediate-stage AMD when compared to a similarly aged human immunodeficiency virus (HIV)-uninfected population (67)

HT	The most frequent cause of hypothyroidism is HT. In a study examining thyroid fine needle aspirates (FNA) and peripheral blood mononuclear cells, HHV-6 DNA prevalence was much higher in HT (82%) than in controls (10%). and viral load was significantly increased in FNA from HT patients, and thyrocytes from HT FNA displayed a 100-fold higher HHV-6 DNA load compared to infiltrating lymphocytes (68). Variant analysis performed in 10 HT samples showed that all samples harbored HHV-6A. *In vitro* infection of thyrocytes with HHV-6A induces modulation of miRNAs considered markers of autoimmune thyroid disease *in vivo*. These alterations were not seen thyrocytes infected with HHV-6B or HHV-7 (69)	The association of AMD with thyroid function is somewhat controversial. In a study with 356 people with AMD 21% reported to have hypothyroidism compared to 11% of those 9,321 without AMD giving an odds ratio of 2.33 (70). On the other side, in a prospective population-based cohort study with 5,573 participants of age ≥55 years, higher free thyroxine (FT4) values were associated with a higher risk of AMD, odds ratio albeit being very small (1.35 for the highest quartile of FT4 levels) (71). However, participants with AMD at baseline (*N* = 567) were excluded from the study. Interestingly, another study found that thyroxine substitution, which can be considered a surrogate marker for hypothyroidism, also correlated with AMD (72).In a smaller study with 114 patients, an association of AMD with not further specified thyroidopathy was found (73).

MS	There is abundant evidence that HHV-6A has an etiopathologic role in MS [for review, see Ref. (74)]. HHV-6A infects astrocytes *via* the CD46, thereby interfering with complement regulation (13, 14), in much the same way as is proposed here for RPE cells. Furthermore, it has recently been postulated that HHV-6A also interacts with Epstein–Barr virus in the CNS of MS patients leading to B-cell transformation and production of oligoclonal immunoglobulins that are typical for MS (12)	Although there are no epidemiologic studies directly examining a possible association between MS and AMD, ocular pathology has been studied in MS patients (75). MS cases with variable clinical severity demonstrated evidence of retinal atrophy and prominent inflammation

## Conclusion

Despite the fact that more and more molecular and genetic mechanisms involved in the pathogenesis of AMD are known today (76–79), the etiologic trigger of the disease has not been identified so far. Center stage is taken by the CD46 on RPE cells with its regulatory role in complement activation, autophagy, and the chemokine/cytokine network. Since CD46 is also the sole cellular receptor for HHV-6A, one is tempted to speculate that HHV-6A might be the trigger for AMD. Supporting evidence comes from the potential of HHV-6A to interfere with inflammatory mechanisms (62, 80). Indirect evidence comes from epidemiological studies that link HHV-6A-related diseases with AMD (Table 1).

In order to substantiate the hypothesis, several approaches are possible:

- Looking for HHV-6A DNA, viral proteins, and HHV-6A-encoded miRNA in pathology samples of AMD.
- Studying experimental infection of RPE cells with HHV-6A *in vitro*.
- Further epidemiological studies evaluating HHV-6A infection and AMD.

## Author Contributions

The author confirms being the sole contributor of this work and approved it for publication.

## Conflict of Interest Statement

The author declares that the research was conducted in the absence of any commercial or financial relationships that could be construed as a potential conflict of interest.

## References

[1] Saifuddin M Parker CJ Peeples ME Gorny MK Zolla-Pazner S Ghassemi M Role of virion-associated glycosylphosphatidylinositol-linked proteins CD55 and CD59 in complement resistance of cell line-derived and primary isolates of HIV-1. J Exp Med (1995) 182:501–9. 10.1084/jem.182.2.501 7543140 PMC2192116

[2] Spear GT Lurain NS Parker CJ Ghassemi M Payne GH Saifuddin M. Host cell-derived complement control proteins CD55 and CD59 are incorporated into the virions of two unrelated enveloped viruses. Human T cell leukemia/lymphoma virus type I (HTLV-I) and human cytomegalovirus (HCMV). J Immunol (1995) 155:4376–81. 7594597

[3] Kraiczy P Skerka C Kirschfink M Brade V Zipfel PF. Immune evasion of *Borrelia burgdorferi* by acquisition of human complement regulators FHL-1/reconectin and Factor H. Eur J Immunol (2001) 31:1674–84. 10.1002/1521-4141(200106)31:6<1674::AID-IMMU1674>3.0.CO;2-2 11385611

[4] Dalgleish AG Beverley PCL Clapham PR Crawford DH Greaves MF Weiss RA. The CD4 (T4) antigen is an essential component of the receptor for the AIDS retrovirus. Nature (1984) 312:763–7. 10.1038/312763a0 6096719

[5] Klatzmann D Champagne E Chamaret S Gruest J Guetard D Hercend T T-lymphocyte T4 molecule behaves as the receptor for human retrovirus LAV. Nature (1984) 312:767–8. 10.1038/312767a0 6083454

[6] Gordon DL Sadlon TA Wesselingh SL Russell SM Johnstone RW Purcell DF Human astrocytes express membrane cofactor protein (CD46), a regulator of complement activation. J Neuroimmunol (1992) 36:199–208. 10.1016/0165-5728(92)90051-L 1370668

[7] He J McCarthy M Zhou Y Chandran B Wood C. Infection of primary human fetal astrocytes by human herpesvirus 6. J Virol (1996) 70:1296–300. 8551599 10.1128/jvi.70.2.1296-1300.1996 PMC189947

[8] Santoro F Kennedy PE Locatelli G Malnati MS Berger EA Lusso P. CD46 is a cellular receptor for human herpesvirus 6. Cell (1999) 99:817–27. 10.1016/S0092-8674(00)81678-5 10619434

[9] Mori Y Seya T Huang HL Akkapaiboon P Dhepakson P Yamanishi K. Human herpesvirus 6 variant A but not variant B induces fusion from without in a variety of human cells through a human herpesvirus 6 entry receptor, CD46. J Virol (2002) 76:6750–61. 10.1128/JVI.76.13.6750-6761.2002 12050388 PMC136280

[10] Ahlqvist J Donati D Martinelli E Akhyani N Hou J Major EO Complete replication cycle and acquisition of tegument in nucleus of human herpesvirus 6A in astrocytes and in T-cells. J Med Virol (2006) 78:1542–53. 10.1002/jmv.20737 17063514

[11] Maeki T Mori Y Features of human herpesvirus-6A and -6B entry. Adv Virol (2012) 2012:1–6. 10.1155/2012/384069 PMC3485865 23133452

[12] Fierz W. Multiple sclerosis: an example of pathogenic viral interaction? Virol J (2017) 14:42. 10.1186/s12985-017-0719-3 28241767 PMC5330019

[13] Kojima A Iwata K Seya T Matsumoto M Ariga H Atkinson JP Membrane cofactor protein (CD46) protects cells predominantly from alternative complement pathway-mediated C3-fragment deposition and cytolysis. J Immunol (1993) 151:1519–27. 8335944

[14] Ingram G Hakobyan S Robertson NP Morgan BP. Complement in multiple sclerosis: its role in disease and potential as a biomarker. Clin Exp Immunol (2009) 155:128–39. 10.1111/j.1365-2249.2008.03830.x 19040603 PMC2675242

[15] Wong WL Su X Li X Cheung CMG Klein R Cheng C-Y Global prevalence of age-related macular degeneration and disease burden projection for 2020 and 2040: a systematic review and meta-analysis. Lancet Glob Health (2014) 2:e106–16. 10.1016/S2214-109X(13)70145-1 25104651

[16] Lambert NG ElShelmani H Singh MK Mansergh FC Wride MA Padilla M Risk factors and biomarkers of age-related macular degeneration. Prog Retin Eye Res (2016) 54:64–102. 10.1016/j.preteyeres.2016.04.003 27156982 PMC4992630

[17] Nussenblatt RB Ferris F Age-related macular degeneration and the immune response: implications for therapy. Am J Ophthalmol (2007) 144:618–26. 10.1016/j.ajo.2007.06.025 17698021 PMC2744410

[18] Nita M Grzybowski A Ascaso FJ Huerva V Huerva V Age-related macular degeneration in the aspect of chronic low-grade inflammation (pathophysiolo-gical parainflammation). Mediators Inflamm (2014) 2014:930671 10.1155/2014/930671 25214719 PMC4152952

[19] Knickelbein JE Chan C-C Sen HN Ferris FL Nussenblatt RB Inflammatory mechanisms of age-related macular degeneration. Int Ophthalmol Clin (2015) 55:63–78. 10.1097/IIO.0000000000000073 PMC4472429 26035762

[20] Kauppinen A Paterno JJ Blasiak J Salminen A Kaarniranta K. Inflammation and its role in age-related macular degeneration. Cell Mol Life Sci (2016) 73:1765–86. 10.1007/s00018-016-2147-8 26852158 PMC4819943

[21] Mitta VP Christen WG Glynn RJ Semba RD Ridker PM Rimm EB C-reactive protein and the incidence of macular degeneration: pooled analysis of 5 cohorts. JAMA Ophthalmol (2013) 131:507. 10.1001/jamaophthalmol.2013.2303 23392454 PMC3625501

[22] Ardeljan D Chan CC. Aging is not a disease: distinguishing age-related macular degeneration from aging. Prog Retin Eye Res (2013) 37:68–89. 10.1016/j.preteyeres.2013.07.003 23933169 PMC3830684

[23] Chen M Xu H. Parainflammation, chronic inflammation, and age-related macular degeneration. J Leukoc Biol (2015) 98:713–25. 10.1189/jlb.3RI0615-239R 26292978 PMC4733662

[24] Franceschi C Garagnani P Vitale G Capri M Salvioli S Inflammaging and “Garb-aging.” Trends Endocrinol Metab (2017) 28:199–212. 10.1016/j.tem.2016.09.005 27789101

[25] Franceschi C Campisi J. Chronic inflammation (inflammaging) and its potential contribution to age-associated diseases. J Gerontol A Biol Sci Med Sci (2014) 69(Suppl 1):S4–9. 10.1093/gerona/glu057 24833586

[26] Gallenga CE Parmeggiani F Costagliola C Sebastiani A Gallenga PE. Inflammaging: should this term be suitable for age related macular degeneration too? Inflamm Res (2014) 63:105–7. 10.1007/s00011-013-0684-2 24202618

[27] Kawa MP Machalinska A Roginska D Machalinski B. Complement system in pathogenesis of AMD: dual player in degeneration and protection of retinal tissue. J Immunol Res (2014) 2014:483960. 10.1155/2014/483960 25276841 PMC4168147

[28] McHarg S Clark SJ Day AJ Bishop PN. Age-related macular degeneration and the role of the complement system. Mol Immunol (2015) 67:43–50. 10.1016/j.molimm.2015.02.032 25804937

[29] Klein RJ Zeiss C Chew EY Tsai JY Sackler RS Haynes C Complement factor H polymorphism in age-related macular degeneration. Science (2005) 308:385–89. 10.1126/science.1109557 15761122 PMC1512523

[30] Haines JL Hauser MA Schmidt S Scott WK Olson LM Gallins P Complement factor H variant increases the risk of age-related macular degeneration. Science (2005) 308:419–21. 10.1126/science.1110359 15761120

[31] Edwards AO Ritter R III Abel KJ Manning A Panhuysen C Farrer LA. Complement factor H polymorphism and age-related macular degeneration. Science (2005) 308:421–24. 10.1126/science.1110189 15761121

[32] Gemenetzi M Lotery AJ Complement pathway biomarkers and age-related macular degeneration. Eye (2016) 30:1–14. 10.1038/eye.2015.203 26493033 PMC4709545

[33] Liao X Lan CJ Cheuk IWY Tan QQ Four complement factor H gene polymorphisms in association with AMD: a meta-analysis. Arch Gerontol Geriatr (2016) 64:123–9. 10.1016/j.archger.2016.01.011 26852301

[34] Francis PJ Zhang H Dewan A Hoh J Klein ML. Joint effects of polymorphisms in the HTRA1, LOC387715/ARMS2, and CFH genes on AMD in a Caucasian population. Mol Vis (2008) 14:1395–400. 18682806 PMC2493023

[35] Micklisch S Lin Y Jacob S Karlstetter M Dannhausen K Dasari P Age-related macular degeneration associated polymorphism rs10490924 in ARMS2 results in deficiency of a complement activator. J Neuroinflammation (2017) 14:4. 10.1186/s12974-016-0776-3 28086806 PMC5234120

[36] Liszewski MK Post TW Atkinson JP. Membrane cofactor protein (MCP or CD46): newest member of the regulators of complement activation gene cluster. Annu Rev Immunol (1991) 9:431–55. 10.1146/annurev.iy.09.040191.002243 1910685

[37] Fagerness JA Maller JB Neale BM Reynolds RC Daly MJ Seddon JM. Variation near complement factor I is associated with risk of advanced AMD. Eur J Hum Genet (2009) 17:100–4. 10.1038/ejhg.2008.140 18685559 PMC2985963

[38] Vogt SD Curcio CA Wang L Li C-M McGwin G Medeiros NE Retinal pigment epithelial expression of complement regulator CD46 is altered early in the course of geographic atrophy. Exp Eye Res (2011) 93:413–23. 10.1016/j.exer.2011.06.002 21684273 PMC3202648

[39] McLaughlin BJ Fan W Zheng JJ Cai H Del Priore LV Bora NS Novel role for a complement regulatory protein (CD46) in retinal pigment epithelial adhesion. Investig Opthalmol Vis Sci (2003) 44:3669 10.1167/iovs.02-0813 12882822

[40] Lyzogubov VV Bora PS Wu X Horn LE de Roque R Rudolf XV The complement regulatory protein CD46 deficient mouse spontaneously develops dry-type age-related macular degeneration-like phenotype. Am J Pathol (2016) 186:1–17. 10.1016/j.ajpath.2016.03.021 PMC4973660 27295359

[41] Kaarniranta K Autophagy – hot topic in AMD. Acta Ophthalmol (2010) 88:387–8. 10.1111/j.1755-3768.2009.01840.x 20353515

[42] Mitter SK Rao HV Qi X Cai J Sugrue A Dunn WA Autophagy in the retina: a potential role in age-related macular degeneration. Adv Exp Med Biol (2012) 723:83–90. 10.1007/978-1-4614-0631-0_12 22183319 PMC3638001

[43] Kaarniranta K Tokarz P Koskela A Paterno J Blasiak J Autophagy regulates death of retinal pigment epithelium cells in age-related macular degeneration. Cell Biol Toxicol (2017) 33:113–28. 10.1007/s10565-016-9371-8 27900566 PMC5325845

[44] Green YA Ben-Yaakov K Adir O Pollack A Dvashi Z. TAK1 is involved in the autophagy process in retinal pigment epithelial cells. Biochem Cell Biol (2016) 94:188–96. 10.1139/bcb-2015-0120 26928052

[45] Dvashi Z Green Y Pollack A. TAK1 inhibition accelerates cellular senescence of retinal pigment epithelial cells. Investig Ophthalmol Vis Sci (2014) 55:5679–86. 10.1167/iovs.14-14349 25118260

[46] Joubert P-E Meiffren G Grégoire IP Pontini G Richetta C Flacher M Autophagy induction by the pathogen receptor CD46. Cell Host Microbe (2009) 6:354–66. 10.1016/j.chom.2009.09.006 19837375

[47] Tuo J The involvement of sequence variation and expression of CX3CR1 in the pathogenesis of age-related macular degeneration. FASEB J (2004) 18:1297–9. 10.1096/fj.04-1862fje 15208270 PMC1971128

[48] Anastasopoulos E Kakoulidou A Coleman AL Sinsheimer JS Wilson MR Yu F Association of sequence variation in the CX3CR1 gene with geographic atrophy age-related macular degeneration in a Greek population. Curr Eye Res (2012) 37:1148–55. 10.3109/02713683.2012.705413 22816662

[49] Ma B Dang G Yang S Duan L Zhang Y. CX3CR1 polymorphisms and the risk of age-related macular degeneration. Int J Clin Exp Pathol (2015) 8:9592–6. 26464724 PMC4583956

[50] Combadière C Feumi C Raoul W Keller N Rodéro M Pézard A CX3CR1-dependent subretinal microglia cell accumulation is associated with cardinal features of age-related macular degeneration. J Clin Invest (2007) 117:2920–8. 10.1172/JCI31692 17909628 PMC1994614

[51] Crane IJ Kuppner MC McKillop-Smith S Knott RM Forrester JV. Cytokine regulation of RANTES production by human retinal pigment epithelial cells. Cell Immunol (1998) 184:37–44. 10.1006/cimm.1997.1235 9626333

[52] Santoro F Greenstone HL Insinga A Liszewski MK Atkinson JP Lusso P Interaction of glycoprotein H of human herpesvirus 6 with the cellular receptor CD46. J Biol Chem (2003) 278:25964–9. 10.1074/jbc.M302373200 12724329

[53] Pedersen SM Höllsberg P. Complexities in human herpesvirus-6A and -6B binding to host cells. Virology (2006) 356:1–3. 10.1016/j.virol.2006.07.028 16959284

[54] Schneider-Schaulies J Cellular receptors for viruses: links to tropism and pathogenesis. J Gen Virol (2000) 81:1413–29. 10.1099/0022-1317-81-6-1413 10811925

[55] Schnorr J Dunster LM Nanan R Schneider-Schaulies J Schneider-Schaulies S ter Meulen V. Measles virus-induced down-regulation of CD46 is associated with enhanced sensitivity to complement-mediated lysis of infected cells. Eur J Immunol (1995) 25:976–84. 10.1002/eji.1830250418 7737301

[56] Murakami Y Tanimoto K Fujiwara H An J Suemori K Ochi T Human herpesvirus 6 infection impairs toll-like receptor signaling. Virol J (2010) 7:91. 10.1186/1743-422X-7-91 20459723 PMC2874541

[57] Isegawa Y Ping Z Nakano K Sugimoto N Yamanishi K. Human herpesvirus 6 open reading frame U12 encodes a functional beta-chemokine receptor. J Virol (1998) 72:6104–12. 9621074 10.1128/jvi.72.7.6104-6112.1998 PMC110416

[58] Dagna L Pritchett JC Lusso P. Immunomodulation and immunosuppression by human herpesvirus 6A and 6B. Future Virol (2013) 8:273–87. 10.2217/fvl.13.7 24163703 PMC3806647

[59] Milne RSB Mattick C Nicholson L Devaraj P Alcami A Gompels UA RANTES binding and down-regulation by a novel human herpesvirus-6 chemokine receptor. J Immunol (2000) 164:2396–404. 10.4049/jimmunol.164.5.2396 10679075

[60] Clark D. Human herpesvirus 6. Rev Med Virol (2000) 10:155–73. 10.1002/(SICI)1099-1654(200005/06)10:3<155::AID-RMV277>3.0.CO;2-6 10815027

[61] Ablashi D Agut H Alvarez-Lafuente R Clark DA Dewhurst S DiLuca D Classification of HHV-6A and HHV-6B as distinct viruses. Arch Virol (2014) 159:863–70. 10.1007/s00705-013-1902-5 24193951 PMC4750402

[62] Agut H Bonnafous P Gautheret-Dejean A. Update on infections with human herpesviruses 6A, 6B, and 7. Med Mal Infect (2017) 47:83–91. 10.1016/j.medmal.2016.09.004 27773488

[63] Knox KK Carrigan DR. Active HHV-6 infection in the lymph nodes of HIV-infected patients: in vitro evidence that HHV-6 can break HIV latency. J Acquir Immune Defic Syndr Hum Retrovirol (1996) 11:370–8. 10.1097/00042560-199604010-00007 8601223

[64] Bates M Monze M Bima H Kapambwe M Clark D Kasolo FC Predominant human herpesvirus 6 variant A infant infections in an HIV-1 endemic region of Sub-Saharan Africa. J Med Virol (2009) 81:779–89. 10.1002/jmv.21455 19319952

[65] Knox KK Carrigan DR. Active human herpesvirus (HHV-6) infection of the central nervous system in patients with AIDS. J Acquir Immune Defic Syndr Hum Retrovirol (1995) 9:69–73. 7712236

[66] Qavi HB Green MT Lewis DE Hollinger FB Pearson G Ablashi DV HIV-1 and HHV-6 antigens and transcripts in retinas of patients with AIDS in the absence of human cytomegalovirus. Investig Ophthalmol Vis Sci (1995) 36:2040–7. 7657542

[67] Jabs DA Van Natta ML Sezgin E Pak JW Danis R Studies of the Ocular Complications of AIDS Research Group. Prevalence of intermediate-stage age-related macular degeneration in patients with acquired immunodeficiency syndrome. Am J Ophthalmol (2015) 159:1115–22.e1. 10.1016/j.ajo.2015.01.037 25769246 PMC6126535

[68] Caselli E Zatelli MC Rizzo R Benedetti S Martorelli D Trasforini G Virologic and immunologic evidence supporting an association between HHV-6 and Hashimoto’s thyroiditis. PLoS Pathog (2012) 8:2–11. 10.1371/journal.ppat.1002951 PMC3464215 23055929

[69] Caselli E D’Accolti M Soffritti I Zatelli MC Rossi R Degli Uberti E HHV-6A in vitro infection of thyrocytes and T cells alters the expression of miRNA associated to autoimmune thyroiditis. Virol J (2017) 14:3. 10.1186/s12985-016-0672-6 28081700 PMC5234148

[70] Bromfield S Keenan J Jolly P McGwin G. A suggested association between hypothyroidism and age-related macular degeneration. Curr Eye Res (2012) 37:549–52. 10.3109/02713683.2011.647223 22577773

[71] Chaker L Buitendijk GHS Dehghan A Medici M Hofman A Vingerling JR Thyroid function and age-related macular degeneration: a prospective population-based cohort study – the Rotterdam Study. BMC Med (2015) 13:94 10.1186/s12916-015-0329-0 25903050 PMC4407352

[72] Gopinath B Liew G Kifley A Mitchell P. Thyroid dysfunction and ten-year incidence of age-related macular degeneration. Investig Ophthalmol Vis Sci (2016) 57:5273–7. 10.1167/iovs.16-19735 27716857

[73] Chatziralli I Mitropoulos PG Niakas D Labiris G Thyroidopathy and age-related macular degeneration: is there any correlation. Biomed Hub (2017) 2:1–1. 10.1159/000454706 PMC6945932 31988898

[74] Leibovitch EC Jacobson S. Evidence linking HHV-6 with multiple sclerosis: an update. Curr Opin Virol (2014) 9:127–33. 10.1016/j.coviro.2014.09.016 25462444 PMC4269240

[75] Green AJ McQuaid S Hauser SL Allen IV Lyness R. Ocular pathology in multiple sclerosis: retinal atrophy and inflammation irrespective of disease duration. Brain (2010) 133:1591–601. 10.1093/brain/awq080 20410146 PMC2877904

[76] Kinnunen K Petrovski G Moe MC Berta A Kaarniranta K. Molecular mechanisms of retinal pigment epithelium damage and development of age-related macular degeneration. Acta Ophthalmol (2012) 90:299–309. 10.1111/j.1755-3768.2011.02179.x 22112056

[77] Ambati J Fowler BJ. Mechanisms of age-related macular degeneration. Neuron (2012) 75:26–39. 10.1016/j.neuron.2012.06.018 22794258 PMC3404137

[78] Parmeggiani F Sorrentino FS Romano MR Costagliola C Semeraro F Incorvaia C Mechanism of inflammation in age-related macular degeneration: an up-to-date on genetic landmarks. Mediators Inflamm (2013) 2013:435607. 10.1155/2013/435607 24369445 PMC3863457

[79] Fritsche LG Fariss RN Stambolian D Abecasis GR Curcio CA Swaroop A. Age-related macular degeneration: genetics and biology coming together. Annu Rev Genomics Hum Genet (2014) 15:151–71. 10.1146/annurev-genom-090413-025610 24773320 PMC4217162

[80] Lusso P. HHV-6 and the immune system: mechanisms of immunomodulation and viral escape. J Clin Virol (2006) 37:S4–10. 10.1016/S1386-6532(06)70004-X 17276368

